# Understanding c-di-GMP-mediated regulation of flagellar motility, one step at a time

**DOI:** 10.1128/jb.00063-26

**Published:** 2026-02-18

**Authors:** George A. O’Toole

**Affiliations:** 1 Department of Microbiology and Immunology, Geisel School of Medicine at Dartmouth 12285 , Hanover, New Hampshire, USA; National Institutes of Health, Bethesda, Maryland, USA

**Keywords:** commentary, cdG, motility, stators, motors

## Abstract

c-di-GMP-mediated control of bacterial motility has been studied in some depth, but many questions remain. N. Bhattarai, W. Guo, J. D. Partridge, R. M. Harshey (J Bacteriol 207:e00353-25, 2025, https://doi.org/10.1128/jb.00353-25) review the current state of the field, present a new 2-step model for the control of flagellar motility, and test the first step of this model. Their model incorporates a plethora of data from different labs, including new structural insights of flagellar motor structure, to test and present a compelling model of by YcgR::c-di-GMP-mediated motility control.

## COMMENTARY

One of the key roles for c-di-GMP is the control of flagellar motility, especially in the context of regulating the transition of motility to sessility during the formation of bacterial biofilms (1). The second messenger c-di-GMP can exert its effects at multiple levels, including transcriptional control of flagellar gene expression and by controlling the function of the flagellar motor (1). A recent publication in the *Journal of Bacteriology* by Bhattarai, Harshey et al., titled “Texas 2-step: a new model for YcgR::c-di-GMP action at the flagellar motor” (2), addresses how the complex formed by the c-di-GMP-binding PilZ domain protein YcgR and the cyclic dinucleotide c-di-GMP impacts flagellar function in *Escherichia coli*. Beyond the key findings of the paper, which I highlight below, is the somewhat unique way the manuscript is formatted—the authors propose a two-step model for YcgR•c-di-GMP control of flagellar motility and provide data to test the first aspect of their 2-part model. As a reader, the authors nicely frame the current state of the field, including the presentation of the key data and the relevant models explaining control of flagellar motility via this protein-second messenger complex. After the reader has absorbed this text, they are fully able to understand the experiments used to test the model.

The key observation here, based on a variety of approaches, is that YcgR•c-di-GMP complex interacts with the stator and/or motor to confer a counterclockwise (CCW) bias on, and reduce rotation of, the motor. The 2-part model presented by the authors is based on some recent structural work indicating that there are large structural changes in the flagellar motor and in the positioning of the stators when switching between clockwise (CW) and CCW rotation (3–6). The challenge, as described by the authors, has been that various models implicate interactions with the stator, the rotor, or both in the proposed mechanisms of YcgR•c-di-GMP function. Here, the authors propose a new “Texas 2-step model,” which, as they state, is named “after the country dance in which partners move smoothly in a CCW arc with quick steps followed by slow ones.” Their Texas 2-step model is in two parts:

1. The YcgR•c-di-GMP complex binds to MotA (part of the MotAB stator) when MotA is in the CCW conformation and being displaced from the C-ring of the flagellar motor; then,
2. MotA facilitates the delivery of YcgR•c-di-GMP to the switch protein FliG, which reduces rotation of the motor.

In this manuscript, the authors test the first part of their model. The authors note, based on previous work, that the CCW rotor bias and reduction of speed can occur sequentially, indicating a “2-step” process of flagellar shut down (7). In the CCW orientation, the stator is located on the outside face of the C-ring (3–6) and thus accessible to YcgR•c-di-GMP binding. A clear prediction of the model is that CW-locked motors should show no (or reduced) impact of the YcgR•c-di-GMP complex, given that CW rotation of the motor requires the stator to be in a position that does not allow easy access by YcgR•c-di-GMP (a point nicely illustrated in two figures in the publication). Using two different strain backgrounds with mutants that favor or lock cells in a CW conformation showed that, indeed, flagellar rotation was much less susceptible to YcgR•c-di-GMP function compared to motors rotating in a CCW direction.

To be philosophical for a moment, what have we learned here, in addition to advancing our better understanding of the control of flagellar rotation in response to c-di-GMP? First, for complex biological systems, it often requires multiple complementary approaches to gain true insight into function. Genetic studies provided the first information that the YcgR•c-di-GMP complex is important for motility control. Subsequent structural studies gave us a picture of the motor/stators, but it is only in the context of additional biochemical and genetic experiments that a more comprehensive picture of motility control could be uncovered. Together, these studies from multiple labs were necessary to get a wholistic view of the mechanism detailed here. And it is not clear to me that all the sequencing in the world would provide such insight. Second, some may have noticed a rise in what I refer to as “science story time,” or the tendency to present nice, tidy reports that purport to have answered all the questions. But most science is not tidy. Here, Harshey and colleagues present data, some of it contradictory on its face, outline a testable model based on these data, and test it. How refreshing. I wish more papers were written this way.
